# Adequacy and implications of antimicrobial prophylaxis for elective surgeries in a tertiary hospital: a cross sectional and retrospective cohort study (ADEQUAP)

**DOI:** 10.1186/s13756-025-01601-x

**Published:** 2025-07-06

**Authors:** Marco Piscaglia, Dolores Martín Sierra, Antonio Huelva Millán, Maria Teresa Garcia Poo, Jesús Rodríguez Baño, Maria Dolores del Toro López

**Affiliations:** 1 Infectious Diseases Department, Luigi Sacco University Hospital, via G.B. Grassi 74, Milan, 20157 Italy; 2 Infectious Diseases and Microbiology Clinical Unit, Virgen Macarena University Hospital, Av. Dr. Fedriani, 3, Seville, 41009 Spain; 3 Department of Medicine, University of Seville/Biomedicine Institute of Seville (IBiS)/CSIC, Seville, Spain

**Keywords:** Surgical antimicrobial prophylaxis, Surgical site infection, Nosocomial infection, Elective surgical procedures, Antimicrobial stewardship program

## Abstract

**Background:**

Surgical antimicrobial prophylaxis (SAP) is essential for preventing surgical site infections (SSI) but is often improperly administered. This study assessed SAP adequacy and its association with SSI and other nosocomial infections (NI) to identify areas for improvement.

**Methods:**

This cross-sectional and retrospective cohort study included adults undergoing elective cardiovascular, orthopaedic, colorectal surgeries and cystectomy in 2022 at a teaching hospital. SAP was considered adequate if it met all local guideline criteria: indication, drug, dose, timing of administration, redosing and duration. Associations between SAP adequacy and SSI were analyzed using generalized mixed models.

**Results:**

Among 723 patients included (median age 68 years; 57.7% male), 714 (98.8%) received SAP. Overall multidomain adherence to SAP guidelines was 34.6%, with high compliance for regimen (92.8%), dose (97.5%), and timing (98.3%), but lower compliance for redosing (63.4%) and duration (53.9%). Regimen adequacy was significantly lower in patients with beta-lactam allergies (55.6% vs. 94.8%, *p* < 0.001) and in cystectomy cases compared to other procedures (41.2% vs. 94.1%, *p* < 0.001). Non-compliant regimens were independently associated with a higher SSI rate (adjusted OR 3.4; 95% CI: 1.8–8.3; *p* = 0.003), but not with non-SSI NIs. Inadequate SAP was also associated with a length of stay (LOS) exceeding 10 days (RR 4.61; *p* < 0.001) and higher 90-day mortality (RR 3.37; *p* = 0.007). Patients who developed an SSI were significantly more likely to develop additional non-SSI NIs (adjusted OR 6.1; 95% CI: 2.8–13.4; *p* < 0.001). Median LOS was longer in patients with SSI (16.5 vs. 7 days, *p* < 0.001), and SSI was also associated with increased 90-day mortality (14.7% vs. 2.7%; RR 5.42; *p* < 0.001).

**Conclusion:**

Non-adherence to SAP guidelines was associated with an increased risk of SSI, prolonged LOS, and greater crude mortality. Key areas for improvement include regimen selection, appropriate redosing, and limiting SAP duration. Patients with beta-lactam allergies were specially at risk of receiving inadequate SAP. Although SAP non-compliance was not independently associated with other NIs, SSIs significantly increased their occurrence.

**Supplementary information:**

The online version contains supplementary material available at 10.1186/s13756-025-01601-x.

## Background

Despite advances in knowledge and prevention, surgical site infection (SSIs) remains the second leading cause of healthcare-related infection in European countries [1, 2]. SSIs are associated with substantial healthcare costs, prolonged hospital stays, rehospitalizations, reoperations, increased mortality rates, and a negative impact on patient´s physical and mental well-being [3]. Prevention of SSIs involves implementing evidence-based interventions during the preoperative, perioperative, and postoperative periods, among which surgical antimicrobial prophylaxis (SAP) is one of the most effective measures, although SAP´s efficacy is significantly diminished if not complemented by other preventive measures [4]. Regardless of recommendations from leading health organizations [1, 2, 5], SAP continues to be inappropriately administered in many hospitals. Common issues include non-compliance with guidelines, incorrect timing of administration, and unnecessary prolongation [6–9]. Non-adherence to SAP guidelines not only increases SSI risk but also contributes to bacterial resistance, adverse drugs effects, and heightened antibiotic consumption up to seven-folds in same cases [10]. Furthermore, up to 60% of surgical patients receive postoperative antibiotics during hospitalization, and nearly 50% are discharged with antibiotics [11]. 

Integrating SAP optimization into antimicrobial stewardship programs (AMS) tailored to local epidemiology and microbial ecology is essential for reducing inappropriate antibiotic use and preventing healthcare-associated infections and antimicrobial resistance.

At the Hospital Universitario Virgen Macarena (HUVM), a surgical site infection surveillance program established in 1996 has enabled the identification of risk situations associated with increased SSI incidence, facilitating feedback to healthcare providers and the implementation of preventive measures. In 2021, the HUVM update its SAP guideline based on newly published national recommendations [12]. Training sessions were conducted for all departments involved in surgical care to ensure widespread dissemination of the updated guidelines.

The primary objective of this study is to evaluate adherence to local SAP guidelines and explore its clinical implications. Specifically, we aimed to assess whether deviations from recommended prophylactic protocols are associated with increased risks of SSIs, other nosocomial infections (NIs), prolonged hospital stay, in-hospital, and 90-day mortality. By identifying specific dimensions and patterns of non-compliance, the study seeks to inform targeted interventions to improve prophylaxis practices and enhance patient safety.

## Materials and methods

### Study design, setting and eligibility

The ADEQUAP study is a cross-sectional analysis aimed at evaluating SAP adequacy and adherence to local guidelines among hospitalized patients undergoing elective surgery. This was followed by a retrospective cohort analysis to examinate SSI rates, associated risk factors, and other outcomes. The study was approved by the local Ethics Committee and conducted in accordance with the World Medical Association´s Declaration of Helsinki and Good Clinical Practice guidelines. The STROBE recommendations were followed for reporting.

The study was conducted at an 800-bed tertiary university hospital from January 1 to December 31, 2022, and included patients aged ≥ 18 years undergoing the following elective procedures: valvular cardiac surgery (including transcatheter aortic valve implantation [TAVI]), aortocoronary bypass, cystectomy, knee arthroplasty, spinal orthopaedic surgery, and colorectal surgery. These procedures were selected based on their inclusion in the hospital’s SSI surveillance program and the high baseline risk of infection. Patients receiving intraoperative antibiotics for reasons other than SAP were excluded.

### Data source, variables and definitions

Patients were identified through the hospital´s surgery registry and SSI surveillance program. Data collected included demographics, length of hospital stay (LOS), beta-lactam allergy status, colonization by multidrug-resistant organism (MDROs), body mass index (BMI), American Society of Anaesthesiologists (ASA) score [13], type of surgery, National Nosocomial Infections Surveillance System (NNIS) index [14], SAP details (antibiotic regimen, dosage, timing, redosing, duration), SSI or other NI occurrence, causative microorganisms, antibiotic treatments, in-hospital and 90-day mortality. Data were extracted from patient records, microbiology laboratory archives, and digital anaesthesiology reports.

The primary outcome was SAP adequacy, defined as adherence to local guidelines regarding antibiotic regimen, timing, administration route, dosage, redosing (for prolonged surgeries or significant blood loss), and duration, while accounting for patient-specific factors such as allergies or colonization by methicillin-resistant *Staphylococcus aureus* (MRSA) or other MDROs. Multi-domain adequacy was defined as compliance with all criteria. Table S1 summarizes the local SAP guidelines. These guidelines were based on the 2021 national consensus by the Spanish Society of Infectious Diseases and Clinical Microbiology (SEIMC) and the Spanish Association of Surgeons (AEC) and adapted to local microbial ecology [12]. *S. aureus* screening and decolonization is routinely performed in all patients undergoing cardiac procedures.

Secondary outcomes included the cumulative incidence of SSI and NIs during hospitalization, as well as in-hospital and 90-day mortality rates. SSIs were classified as superficial, deep, or organ-space infections based on European Centre for Disease Prevention and Control (ECDC) criteria and monitored for 30 days post-surgery (or up to 90 days for surgeries involving implants) [15]. Other NIs were defined based on microbiological criteria or clinical judgement and categorized as bloodstream infections, lower respiratory tract infections, urinary tract infections, or other types.

### Statistical analysis

Qualitative variables were expressed as percentages, and quantitative variables as medians with interquartile ranges (IQRs). One-year cumulative incidence of SSI and NIs was calculated overall and stratified by procedure type and NNIS index. Bivariate analysis of associations between exposure variables and outcomes were conducted using Chi-square or Fisher’s exact tests, with relative risks (RR) and 95% confidence intervals (CI). Multivariable logistic regression models were constructed to assess the risk of SSI and NI. Surgical type was modeled as a random intercept in generalized linear mixed models to account for clustering and heterogeneity across procedure types. Variables with p value < 0.15 in bivariate analysis or deemed clinically relevant were included in the models and selected via a manual stepwise backward procedure, retaining those with p-values < 0.1. Collinearity and interactions between variables were examined when clinically appropriate. Model predictive performance was assessed using area under the receiver operating characteristic (AUROC) curves with 95% CIs.

## Results

After excluding 33 patients who were receiving antibiotic treatment at the time of surgery, a total of 756 patients who underwent the selected procedures during the study period were included in the analysis. Baseline patient characteristics and surgical procedures are summarized in Table 1. Among these patients, 298 (41.2%) underwent cardiac surgery, 236 (32.6%) colorectal surgery, 172 (23.8%) orthopaedic surgery, and 17 (2.4%) cystectomy. Thirty-six (5%) patients reported a beta-lactam allergy, and 1 (0.1%) was colonized by a MDRO.

**Table 1:** Baseline patient characteristics and types of surgery in the study population

Baseline characteristics	*N* = 723
Age (years) (median, IQR)	68 (60–75)
Female sex	306 (42.3)
BMI (kg/m^2^) (median, IQR)	28.92 (25.20–32.5)
Beta-lactam allergy	36 (5)
MRSA colonization	0 (0)
Other MDRO colonization	2 (0.1)
Surgical contamination grade	
* Clean*	472 (65.4)
* Clean-contaminated*	248 (34.3)
* Contaminated*	2 (0.3)
ASA score	
* 1*	11 (1.5)
* 2*	154 (21.3)
* 3*	429 (59.3)
* 4*	129 (17.8)
NNIS score	
* -1*	23 (3.2)
* 0*	164 (22.7)
* 1*	460 (63.6)
* 2*	76 (10.5)
* 3*	0
LOS before surgery (median, IQR)	1 (1–1)
LOS after surgery (median, IQR)	7 (5–12)
Previous surgery	58 (8)
Reoperation	104 (14.4)
90-day all-cause mortality	31 (4.3)
In-hospital mortality	16 (2.2)
Type of surgery	
Cardiovascular	198 (41.2)
* Valvular*	176 (24.3)
* TAVI*	33 (4.6)
* Bypass*	83 (11.5)
* Valvular + Bypass*	6 (0.8)
Cystectomy	17 (2.4)
Orthopaedic	172 (23.8)
*Primary knee arthroplasty*	78 (10.8)
* Revision knee arthroplasty*	33 (4.6)
* Spinal surgery*	61 (8.4)
Colorectal	236 (32.6)

Data are expressed as N (%) except when indicated as median and interquartile range (IQR). BMI: body mass index; MRSA: methicillin-resistant *S. aureus*; MDRO: multidrug-resistant organism; ASA: American Society of Anaesthesiologists; NNIS: National Nosocomial Infections Surveillance System; LOS: length of stay; TAVI: transcatheter aortic valve implantation.

### Surgical antibiotic prophylaxis adequacy

All procedures included in the study had an indication of SAP according to the local guidelines. SAP was administered in 714 (98.8%) patients. Among the 9 patients who did not receive SAP, the omission was considered inadequate across all SAP prescription dimensions. Table 2 provides SAP adequacy by surgical procedure.

**Table 2:** Surgical antibiotic prophylaxis adequacy by surgery type

	CV *N* = 298	CYS *N* = 17	ORTHO *N* = 172	COL *N* = 236	ALL *N* = 723
**Regimen**	279 (93.6)	7 (41.2)	171 (99.4)	214 (90.7)	671 (92.8)
***MIS***	*0*	*0*	*0*	*0*	*0*
**Dose**	295 (99)	16 (94.1)	167 (97.1)	227 (96.2)	705 (97.5)
***MIS***	*0*	*0*	*0*	*0*	*0*
**Timing**	263 (97.8)	15 (93.8)	172 (100)	227 (97.8)	677 (98.3)
***MIS***	*29*	*1*	*0*	*4*	*34*
**Redosing**	141 (55.1)	8 (50)	127 (74.7)	148 (65.1)	424 (63.4)
***MIS***	*42*	*1*	*2*	*9*	*54*
**Duration**	62 (20.8)	14 (84.2)	107 (62.2)	206 (87.3)	390 (53.9)
***MIS***	*0*	*0*	*0*	*0*	*0*
**Multidomain**	41 (15.7)	3 (18.8)	82 (48.2)	108 (46.9)	234 (34.6)
***MIS***	*37*	*1*	*2*	*6*	*46*

Data are expressed as N (%). CV: cardiovascular surgery; CYS: cystectomy; ORTHO: orthopaedic surgery; COL: colorectal surgery; MIS: missing values (N). Percentages do not account for missing data.

The antibiotic regimen was adequate in 671 (92.8%) patients. Among the 47 (90.4%) patients with an inadequate regimen, the prescribed antimicrobial spectrum was narrower than that recommended in the guidelines. Cystectomy showed the highest proportion of inadequate regimens, with 7 out 17 cases (41.2%) being non-compliant. SAP dosage and timing were appropriate in over 93% of patients across all surgery types. Redosing was adequately performed in 424 of 668 (63.4%) patients. Among the 392 patients for whom redosing was indicated, compliance was achieved in 158 cases (40.3%). The lowest adherence among those requiring redosing was observed in general surgery and cystectomy (33.9% and 30%, respectively), while higher compliance was observed in cardiovascular and orthopaedic surgery (44.7% and 45.9%, respectively).

SAP duration was appropriate in 390 (53.9%) patients, with the lowest compliance observed in cardiovascular surgery (62/298, 20.8%). Among cardiac surgery patients with inadequate duration, most received SAP for 24 h postoperatively (226, 75.8%). Overall, multidomain adequacy was achieved in 234 (34.6%) patients, with compliance rates around 50% in orthopaedic and colorectal surgeries, but below 20% in cardiovascular procedures and cystectomy. Patients with beta-lactam allergies were significantly less likely to receive an adequate SAP regimen than non-allergic patients [adequacy: 20 (55.6%) vs. 651 (98.8%), *p* < 0.001]. This difference was more pronounced in clean surgeries (cardiac and orthopaedic) [7 (38.9%) vs. 443 (98%), *p* < 0.001], than in clean-contaminated surgeries (colorectal and cystectomy) [13 (72.2%) vs. 208 (88.5%), *p* = 0.045].

The proportion of patients with inadequate SAP and LOS exceeded 10 days was 37.8% (159/421), compared to 11.6% (27/232) among those with adequate SAP (RR 4.608, 95% CI: 2.950–7.194; *p* < 0.001). In addition, an inadequate regimen was associated with higher 90-day mortality (6/52, 11.5% vs. 25/671, 3.7%; RR 3.367, 95% CI: 1.318–8.620; *p* = 0.007).

### Surgical site infections

A total of 53 patients developed SSI, corresponding to an incidence of 7.3% (95% CI: 5.6–9.4%). Stratified by surgical procedure, SSI incidence was: cardiovascular surgery 4.4% (13/298; 95% CI: 2.5–7.1%), orthopaedic surgery 5.2% (9/172; 95% CI: 2.6–9.3%), colorectal surgery 8.9% (21/236; 95% CI: 5.8–13.0%), and cystectomy 58.8% (10/17; 95% CI: 35.6–79.3%). Table S2 in supplementary materials summarizes SSI incidence by surgical type and NNIS index. Of the 53 SSIs, 6 (11.3%) were superficial, 10 (18.9%) deep, and 37 (69.8%) organ/space infections.

Table 3 presents the bivariate and multivariate analysis of SSI risk factors. Patients with SSI were more frequently male (75.5% vs. 56.3%, *p* = 0.006), had undergone prior surgery (17% vs. 7.3%; *p* = 0.013), had longer surgery duration (215 vs. 165 min, *p* < 0.001), and more often underwent clean-contaminated or contaminated procedures (58.5% vs. 32.7%; *p* < 0.001).

**Table 3:** Bivariate and multivariate analysis of factors associated with SSI

Variable	No-SSI *N* = 670	SSI *N* = 53	*P*	aOR (95% CI)	*P*
**Age > 70 years**	311 (46.4)	24 (45.3)	0.873		
**Male sex**	377 (56.3)	40 (75.5)	0.006	**6.02 (1.60-22.73)**	0.008
**Previous surgery**	49 (7.3)	9 (17)	0.013	**3.05 (1.21–7.38)**	0.013
**BMI > 35**	87 (13)	7 (13.2)	0.963		
**Surgery duration, min** **median, [IQR]**	165 [120–215]	215 [149.5–263]	< 0.001	**1.005 (1.002–1.009)**	0.004
**Type of surgery**					
Clean surgery	451 (67.3)	22 (41.5)	< 0.001		
Clean-contaminated or contaminated surgery	219 (32.7)	31 (58.5)	< 0.001		
**ASA ≥ 2**	520 (77.6)	38 (71.7)	0.323		
**NNIS ≥ 2**	67 (10)	9 (17)	0.111		
**Beta-lactam allergy**	31 (4.6)	5 (9.4)	0.121		
**SAP administered**	662 (98.8)	52 (98.1)	0.661		
**Inadequate SAP regimen**	40 (6)	12 (22.6)	< 0.001	**3.38 (1.80–8.33)**	0.003
**Inadequate SAP timing**	2 (0.3)	1 (2)	0.084		
**Inadequate SAP redosing**	213 (34.9)	23 (46.9)	0.09		
**Inadequate SAP duration**	308 (46.5)	17 (32.7)	0.054		
**Overall SAP adequation**	219 (35.4)	15 (30)	0.438		

Data are expressed in N (%), except when indicated as median (IQR). Generalized mixed model adjusted by surgery type. SAP: surgical antibiotic prophylaxis; SSI: surgical site infections, BMI: body mass index; ASA: American Society of Anaesthesiologists; NNIS: National nosocomial infections surveillance system; aOR: adjusted Odds Ratio; CI: confidence interval. The following variables were considered for inclusion in the multivariable models: age, sex, prior surgery, beta-lactam allergy, BMI > 35, ASA score, clean-contaminated/contaminated surgery, surgery duration, and adherence to regimen SAP.

SSI was more frequent with non-recommended regimens (23.1% vs. 6.1%; RR 3.367, 95% CI: 1.318–8.621; *p* < 0.001), non-compliant timing (2% vs. 0.3%; *p* = 0.084), and omitted redosing when indicated (9.7% vs. 6.1% respectively; *p* = 0.09). In multivariate analysis, adjusting by surgical procedure, independent predictors of SSI included male gender (aOR 6.02; 95% CI: 1.60-22.73), prior surgery (aOR 3.05; 95% CI: 1.21–7.38), longer surgery duration (aOR per minute: 1.005; 95% CI: 1.002–1.009), and inadequate regimen (aOR 3.38; 95% CI: 1.80–8.33).

Patients with SSI had significantly longer median LOS compared to those without SSI (16.5 vs. 7 days, respectively; *p* < 0.001). The proportion of patients with LOS exceeding 10 days was 74% (37/50) among those with SSI, compared to 31.1% (204/656) among those without SSI (RR 6.306; 95% CI: 3.382–12.118; *p* < 0.001). SSI was also associated with increased 90-day mortality (15/102, 14.7% vs. 16/590, 2.72%; RR 5.42; 95% CI: 2.60-11.31; *P* < 0.001).

The etiological agent was identified in 35 (66%) SSIs; 21 (60%) were monomicrobial (Table 4). Gram-positive cocci predominated in clean surgeries (8/14, 57.1%) while gram-negative bacilli were more common in clean-contaminated procedures (21/27, 77.8%). Notably, *Enterobacteriaceae* were isolated in 6 of 13 (46.1%) cardiac SSI cases. The microorganism was susceptible to the administered SAP in 21 of 35 (60%) cases; adherence to guidelines would have increased coverage to 26 of 35 cases (74.3%).

**Table 4:** Etiologic agents of surgical site infections (SSI)

	CV *N* = 13	CYS *N* = 10	ORTHO *N* = 9	COL *N* = 21	ALL *N* = 53
**Monomicrobial**	**8 (61.5)**	**4 (40.0)**	**5 (55.6)**	**4 (19.0)**	**21 (39.6)**
**Polymicrobial**	**2 (15.4)**	**2 (20.0)**	**0 (0)**	**10 (47.6)**	**14 (26.4)**
**No identification**	**3 (23.1)**	**4 (40.0)**	**4 (44.4)**	**7 (33.3)**	**18 (34.0)**
***MSSA***	0 (0)	0 (0)	1 (11.1)	0 (0)	1 (1.9)
***MRSA***	1 (7.7)	0 (0)	0 (0)	0 (0)	1 (1.9)
***CoNS***	3 (23.1)	0 (0)	2 (22.2)	1 (4.8)	6 (11.3)
**Enterococci**	1 (7.7)	1 (10.0)	0 (0)	4 (19.0)	6 (11.3)
**Enterobacterales**	6 (46.1)	4 (40.0)	1 (11.1)	12 (57.1)	23 (43.4)
***P. aeruginosa***	0 (0)	1 (10.0)	1 (11.1)	3 (14.3)	5 (9.4)
**Anaerobic**	0 (0)	0 (0)	0 (0)	6 (28.6)	7 (13.2)
***Candida spp.***	0 (0)	1 (10.0)	0 (0)	0 (0)	1 (1.9)

Data are expressed in N (%). CV: cardiovascular surgery; CYS: cystectomy; ORTHO: orthopaedic; COL: colorectal surgery; *MSSA*: methicillin-sensible *S. aureus*; *MRSA*: methicillin-resistant *S. aureus*; *CoNS*: coagulase-negative staphylococci.

### Other nosocomial infections

A total of 31 (10.9%) patients developed a non-SSI nosocomial infection. Table S3 details infection types by surgical procedure. The most common were urinary tract infections (35/723, 4.8%) and bloodstream infections (32/723, 4.4%). Cystectomy had the highest non-SSI infection rate (6/17, 35.3%), followed by cardiovascular (46/298, 15.4%), colorectal (21/236, 8.9%), and orthopaedic surgery (6/172, 3.5%). Only one case of *Clostridioides difficile* infection was reported. Most non-SSI infections occurred after SSI diagnosis. Bivariate and multivariate analysis of non-SSI risk factors are shown in Table S4. While SAP inadequacy was not independently associated with non-SSI infection, having an SSI was a strong predictor (aOR 6.076; 95% CI: 2.753–13.421; *p* < 0.001).

## Discussion

This study highlights the clinical relevance of SAP adequacy in elective high-risk surgeries. While overall adherence to SAP guidelines was high (particularly regarding regimen selection, dosage, and timing), important gaps remain, notably in intraoperative redosing (especially in cardiovascular and cystectomy procedures), regimen selection for cystectomy, and prolonged prophylaxis in cardiovascular surgery. As seen in prior studies [16–21], patients labeled as beta-lactam allergic had significantly lower adherence rates, despite the existence of validated alternative regimens.

Our results suggest that non-compliance with key SAP elements is associated with increased risk of SSIs, prolonged LOS, other NIs, and higher crude mortality. While the study design limits causal inference, these associations support strengthening SAP adherence as a central component of surgical safety and infection prevention initiatives.

Although SAP adherence has been extensively studied [22–31], relatively few studies have directly linked guideline non-adherence with SSI incidence (Table S5) [32, 33]. Reported compliance varies widely (from 0.3 to 84.5%) depending on local practices, resource availability, and which SAP components are evaluated [34]. In our institution, previous audits had shown high compliance with indication and timing (over 97%) but lower adherence in SAP duration. Following updated guidelines and targeted education, this study undertook a multimodal audit encompassing six SAP dimensions: indication, regimen, dose, timing, redosing, and duration.

Overall multidimensional adherence was only 34.6%, despite > 97% compliance in indication, timing and dose. Cardiovascular and urological surgeries had the lowest full adherence (15.7% and 18.8%, respectively), with particularly poor compliance in regimen selection for cystectomy. This likely reflects limited awareness or confidence in guideline-concordant alternatives among surgical teams.

Redosing compliance was suboptimal across procedures, particularly in cardiovascular (55.1%), urological (50%) and colorectal surgeries (65.1%). Factors contributing to this include the absence of real-time decision support, insufficient awareness of redosing triggers (e.g., operative time, blood loss), and documentation gaps. Prolonged prophylaxis beyond recommended durations was common, especially in cardiovascular (20.8%) and orthopedic procedures (62.2%), possibly due to entrenched practices and perceived complexity or risk in these procedures. These findings point to the need for targeted interventions, such as intraoperative checklists or automated alerts, to support compliance in real time.

Internationally, SAP adherence in high-income countries ranges between 24.6% and 46.8% [30, 31], with significantly lower rates reported in lower-income settings (2–19%) [29, 35] (Table S5). Our data are in line with those from developed centers, which still show room for improvement in redosing and duration [30, 31, 36],. In contrast, regimen and timing remain more problematic in developing countries, with regimen compliance between 4.2% and 37.6% [24, 29], although interventions have demonstrated substantial improvement [32].

Our findings align with previous reports showing reduced SAP adequacy in patients labelled as allergic to beta-lactams [37], particularly in clean surgeries, where adherence in our cohort dropped to 38.9%. Despite institutional protocols and validated alternatives, compliance remains suboptimal, likely due to unfamiliarity with alternatives and prescriber hesitation. Our data indicate that non-guideline regimens (often narrower in spectrum) are associated with higher SSI risk, emphasizing the importance of prescriber support and training in allergy management. Incorporating validated allergy risk stratification tools or point-of-care testing may help expand safe use of first-line agents in low-risk patients.

Although redosing omission was associated with higher SSI rates, statistical significance was limited by sample size and missing data. Factors such as prior surgery and prolonged operative time were associated with higher SSI risk, as previously described [38], as was male gender [39, 40], possibly due to factors like higher bacterial colonization, tobacco use, and obesity. Interestingly, beta-lactam allergy was not associated with increased SSI risk in our study, contrary to some reports [41, 42], though this may be due to limited statistical power.

Previous studies in developed and developing countries have shown that intervention to improve SAP adherence can significantly reduce SSI rates [32, 36]. In Greece, a multicenter intervention study reported an increase in SAP compliance from 28.2 to 43.9% and a drop in SSIs from 6.9 to 4% [36]. In Pakistan, SAP adherence improved from 37.6 to 91%, with SSI rates dropping from 32.7 to 15.2% after implementing the WHO checklist [32]. SSIs contribute substantially to healthcare costs, reduced quality of life, and increased mortality [43]. Like other research findings, in our cohort, both inadequate SAP and the occurrence of SSIs were associated with prolonged LOS and increased mortality [32, 44], supporting the broader clinical and economic importance of high-quality prophylaxis. Furthermore, the link between SSIs and subsequent NIs reinforces the value of robust SSI prevention in reducing downstream infection burden and antibiotic exposure—key goals of antimicrobial stewardship (AMS) programs [45].

Microbiological documentation was achieved in only 66% of SSIs (50% in superficial and deep infections, 76% in organ-space infections), likely limited by prior antibiotic use. Notably, enterobacteria were unexpectedly frequent in cardiac surgery SSIs, consistent with recent ECDC reports [46], while gram-positive pathogens were less common, potentially reflecting local epidemiology influenced by aging populations and decolonization practices. This microbiological pattern may limit generalizability to settings where gram-positive organisms predominate. This finding emphasizes the need for institution-specific surveillance to guide SAP protocols. Although 40% of SSIs were not covered by the administered SAP, adherence to local guidelines would have increased coverage to 74.3%, underscoring the potential value of protocol adherence even in settings of changing pathogen profiles.

This study has several limitations that must be considered when interpreting the results. First, the retrospective and single-center design, as well as the inclusion of a limited number of surgical specialties, restricts the generalizability of our findings, particularly regarding low-frequency outcomes such as SSIs. Additionally, the absence of a comparable pre-intervention cohort limits the ability to assess temporal trends or infer the causal effects of specific interventions or guidelines over time. Second, a degree of data loss occurred due to inconsistent documentation and reliance on operator-dependent variables, such as timing of redosing and intraoperative blood loss. Nonetheless, we sought to mitigate this limitation by selecting procedures under active surveillance, with prospective follow-up and microbiological confirmation, thereby strengthening internal validity. Third, although we applied multivariable adjustments to account for potential confounders, residual confounding due to unmeasured factors (such as surgical technique, intraoperative sterility, or postoperative care practices) cannot be ruled out. The cross-sectional nature of the analysis further limits our ability to establish causal relationships. While shared risk factors may underline the occurrence of both SSIs and other NIs, it is notable that most NIs occurred after an SSI diagnosis, which supports the plausibility of a clinical association between SSIs and subsequent infectious complications.

Finally, while our multidimensional assessment of SAP adherence (including indication, regimen, dose, timing, redosing, and duration) provides a comprehensive overview of practice, the interpretation of these results should remain cautious in light of the methodological constraints outlined above.

Despite these limitations, our study underscores the fact that SAP adherence is uneven across different surgical contexts and across different components of the prophylactic regimen. Although adherence to indication, dose, and timing was high, challenges persist in optimizing redosing and avoiding unnecessary extension of prophylaxis duration. These two components appear particularly vulnerable to non-compliance, likely due to their dependence on intraoperative decisions and entrenched clinical habits. As ongoing randomized controlled trials explore optimal SAP duration in complex surgeries, additional implementation strategies (such as intraoperative decision support tools, real-time AMS interventions, and enhanced prescriber education) are urgently needed. Furthermore, the appropriate selection of prophylactic antibiotics, especially in patients labeled as allergic to beta-lactams, emerges as a critical area for quality improvement with direct impact on SSI prevention. Finally, while pharmacologic interventions are central to SSI prevention, the contribution of non-antibiotic strategies (e.g., surgical technique, asepsis, wound care) remains significant, although more difficult to measure and optimize in clinical practice.

## Conclusions

SAP is a cornerstone of SSI prevention. However, the dissemination of guidelines and training efforts alone have not been sufficient to ensure adequate adherence across all SAP dimensions. Our findings highlight the need for more effective and targeted strategies (such as real-time decision support and multidisciplinary feedback) to improve compliance, particularly regarding intraoperative redosing and prophylaxis duration. Patients labeled as allergic to β-lactams remain at increased risk of receiving inadequate SAP, underscoring the need for improved allergy management protocols. Inadequate SAP was associated with a higher risk of SSIs, which in turn were linked to prolonged hospital stays, increased mortality, and a higher incidence of other nosocomial infections. These findings reinforce the essential role of AMS programs in optimizing SAP practices and minimizing the burden of healthcare-associated infections.

## Electronic supplementary material

Below is the link to the electronic supplementary material.

Supplementary Material 1

## Data Availability

No datasets were generated or analysed during the current study.
