# Supplementary material for: Adequacy and implications of antimicrobial prophylaxis for elective surgeries in a tertiary hospital: a cross sectional and retrospective cohort study (ADEQUAP)
Source: Antimicrob Resist Infect Control. 2025 Jul 6;14:82. doi: 10.1186/s13756-025-01601-x (PMC12232766; doi:10.1186/s13756-025-01601-x)
Supplement: Supplementary file 1 — Supplementary Material 1 [file 13756_2025_1601_MOESM1_ESM.docx]

**Supplementary material**

**Table S1. Summary of local guideline recommendations for Surgical Antibiotic Prophylaxis (SAP)**

| Indication | SAP is recommended in clean-contaminated and select clean procedures, such as orthopaedic and cardiac surgeries. SAP is not indicated in the presence of an active infection, where therapeutic antibiotic treatment is required instead. | | |
| --- | --- | --- | --- |
| Timing | SAP should be administered within 120 minutes prior to incision, ideally just before anesthetic induction. For short half-life beta-lactams, such as cefazolin, administration within 60 minutes pre-incision is recommended. In surgeries involving limb ischemia, SAP should be administered before inflating the tourniquet. | | |
| Duration | For most surgical procedures, a single dose of an antibiotic with a half-life sufficient to maintain effective serum and tissue drug concentrations throughout the surgery is appropriate. For cardiac surgeries, SAP may be extended up to 24 hours postoperatively. | | |
| Redosing | An additional intraoperative dose is recommended if the procedure duration exceeds twice the antibiotic half-life. For cefazolin or similar short half-life antibiotics, a second intraoperative dose should be administered at 3 hours. Redosing is also indicated in cases accelerate antibiotic clearance (e.g., burns, very high glomerular filtration rates) or significant intraoperative blood loss (>1500 mL in adults). For procedures involving ischemia, an additional dose should be administered after restoring blood flow. | | |
| Regimen | The preferred antibiotic regimen, along with alternatives for patients with allergies or MRSA colonization, is summarized as follows: | | |
|  | Clean surgery | Preferred:  Cefazolin 2g IV | Alternative:  Teicoplanin 800 mg + gentamicin 3mg/kg IV |
|  | Clean-contaminated surgery | Preferred:  Amoxicillin/Clavulanic Acid 2 g + gentamicin 3mg/kg IV | Alternative: Gentamicin 3 mg/kg + metronidazole 500mg IV. |
| SAP: surgical antibiotic prophylaxis; IV: intravenous. | | | |

**Table S2. Surgical site infections rates by type of surgery and stratified by NNIS class.**

| NNIS class | CV  N = 298 | CYS  N = 17 | ORTHO  N = 172 | COL  N = 236 | ALL  N = 723 |
| --- | --- | --- | --- | --- | --- |
| -1 | 0/2 (0) | 0/0 (0) | 0/0 (0) | 1/21 (4.8) | 1/23 (4.3) |
| 0 | 1/13 (7.7) | 3/6 (50) | 2/33 (6.1) | 7/112 (6.3) | 13/164 (13) |
| 1 | 10/264 (3.8) | 7/11 (63.6) | 4/99 (4) | 9/86 (10.5) | 30/460 (6.5) |
| 2 | 2/19 (10.5) | 0/0 (0) | 3/40 (7.5) | 4/17 (23.5) | 9/76 (11.8) |
| 3 | 0/0 (0) | 0/0 (0) | 0/2 (0) | 0/0 (0) | 0/2 (0) |
| Total | **13/298 (4.4)** | **10/17 (58.8)** | **9/172 (5.2)** | **21/236 (8.9)** | **53/723 (7.3)** |
| Data are expressed as N (%). NNIS: National Nosocomial Infections Surveillance System; CV: cardiovascular surgery; CYS: cystectomy; ORTHO: orthopaedic; COL: colorectal surgery. | | | | | |

**Table S3. Nosocomial infections non-SSI by type of surgery**

|  | CV  N = 298 | CYS  N = 17 | ORTHO  N = 172 | COL  N = 236 | ALL  N = 723 |
| --- | --- | --- | --- | --- | --- |
| No-SSI HAI | **46 (15.4)** | **6 (35.3)** | **6 (3.5)** | **21 (8.9)** | **79 (10.9)** |
| RTI | 12 (4) | 1 (5.9) | 0 (0) | 2 (0.8) | 15 (2.1) |
| BSI | 15 (5) | 4 (23.5) | 0 (0) | 12 (5.1) | 32 (4.4) |
| UTI | 24 (8.1) | 2 (11.8) | 4 (2.3) | 5 (2.1) | 35 (4.8) |
| Others | 5 (1.7) | 3 (17.7) | 2 (1.1) | 6 (2.5) | 16 (2.2) |
| Data are expressed in N (%). CV: cardiovascular surgery; CYS: cystectomy; ORTHO: orthopaedic; COL: colorectal surgery; SSI: surgical site infections; HAI: healthcare-associated infections; RTI: respiratory tract infection; BSI: bloodstream infection; UTI: urinary tract infection; aOR: adjusted Odds Ratio; CI: confidence interval. | | | | | |

**Table. S4 Bivariate and multivariate analysis of factors associated with Nosocomial Infection (NI) Non-SSI.**

| Variable | No NI  N=644 | NI  N=79 | *P* | aOR (95% CI) | *P* |
| --- | --- | --- | --- | --- | --- |
| **Age > 70 years** | 289 (44.9) | 46 (58.2) | 0.025 | 1.694  (0.935- 3.067) | 0.082 |
| **Male sex** | 362 (56.2) | 55 (69.6) | 0.023 |  |  |
| **Previous surgery** | 50 (7.8) | 8 (10.1) | 0.466 |  |  |
| **BMI > 35** | 87 (13.5) | 7 (8.9) | 0.246 |  |  |
| **Surgery duration, min**  **median, [IQR]** | 165 [120-215] | 195 [155-267] | <0.001 |  |  |
| **Clean surgery** | 421 (65.4) | 52 (65.8) | 0.937 |  |  |
| **Clean-contaminated or contaminated surgery** | 223 (34.6) | 27 (34.2) | 0.937 |  |  |
| **ASA ≥ 3** | 491 (76.2) | 67 (84.8) | 0.087 | 3.201  (1.234- 8.298) | 0.017 |
| **NNIS ≥ 2** | 64 (9.9) | 12 (15.2) | 0.151 |  |  |
| **Beta-lactam allergy** | 33 (5.1) | 3 (3.8) | 0.609 |  |  |
| **Pre-intervention days > 5** | 85 (13.2) | 15 (19) | 0.160 |  |  |
| **Inadequate SAP regimen** | 41 (6.4) | 11 (13.9) | 0.014 | 1.623  (0.649- 4.065) | 0.3 |
| **Inadequate SAP timing** | 3 (0.5) | 0 (0) | 0.547 |  |  |
| **Inadequate SAP redosing** | 203 (34.2) | 33 (50) | 0.011 |  |  |
| **Inadequate SAP duration** | 282 (44.3) | 42 (53.8) | 0.111 |  |  |
| **Overall SAP adequation** | 218 (36.3) | 16 (6.8) | 0.044 |  |  |
| **SSI** | 34 (5.3) | 19 (24.1) | <0.001 | 6.076  (2.753- 13.421) | <0.001 |
| Data are expressed as N (%), except when indicated as median (IQR). Generalized mixed model adjusted by surgery type. SSI: surgical site infections. BMI: body mass index. ASA: American Society of Anaesthesiologists. NNIS: National Nosocomial Infections Surveillance System. aOR: adjusted Odds Ratio; CI: confidence interval. | | | | | |

**Table S5. Studies regarding the appropriateness of SAP and its relationship with SSI occurrence.**

| Study | Setting | Population | Intervention | Comparison | Outcomes |
| --- | --- | --- | --- | --- | --- |
| Goede et al. 2013 (31) | USA, 1 hospital, 9 different surgeries | N = 760 | Cross-sectional study  Evaluation of regimen, dose, timing, redosing. | Adherence to local guidelines in patients who developed SSI. | Regimen 89.2%  Redosing: 54.9%  Overall: 24.6%  Remaining outcomes expressed by drug. |
| Morioka et al. 2022 (32) | Japan, 16 hospitals, 18 different surgeries | N = 688 | Cross-sectional study  Evaluation of regimen, timing, redosing, duration. | Adherence to national guidelines. | Regimen 78.5%  Timing 96%  Redosing 91.6%  Duration: 61.4%  Overall: 46.8%  Overall, by surgery:  CIED implantation: 8%  Tooth extraction: 10%  Coronary artery bypass graft: 20.8% |
| Celik et al.2024 (36) | Turkey, 47 hospitals | N = 7978 | Multicenter cross-sectional survey.  Evaluation of regimen, timing, duration. | Adherence to national guidelines | Regimen 75.5%  Timing 66.4  Duration: 50.7%  Overall: 19% |
| Enriquez et al. 2022 (24) | Liberia, 1 hospital, 2 surgeries | N = 143 | Cross-sectional study  Evaluation of timing. | Adherence to international guidelines | Timing: 20.3% |
| Khan et al. 2022 (25) | Pakistan, 2 hospitals, 3 general surgical procedures | N = 660 | Cross-sectional study  Evaluation of regimen, timing. | Adherence to international guidelines | Regimen: 4.2%  Timing: 51% |
| Musmar et al. 2014 (30) | Palestine, 3 hospitals, 3 surgeries | N = 400 | Prospective study  Evaluation of regimen, timing and duration in elective and emergent procedures. | Adherence to international guidelines | Regimen: 18.5%  Timing: 59.8%  Duration: 31.8%  Overall: 2% |
| Chorafa et al. 2021 (37) | Greece, 3 hospitals, 6 surgeries | N = 1447 (768 vs 679) | Multimodal intervention based on education, audit and feedback.  Evaluation of  regimen, timing, duration, and SSI incidence. | Adherence to local and national guidelines and SSI incidence before and after intervention. | Regimen: 89.6 *vs.* 96.3%  Timing: 78.1 *vs*. 74.9%  Duration: 33.7 *vs* 60.3%  Overall: 28.2 *vs* 43.9%  SSI incidence: 6.9 *vs* 4%. |
| Toor et al. 2015 (33) | Pakistan, 1 hospital, 6 surgeries | N = 613 (303 vs 310) | Implementation of WHO surgical safety checklist.  Evaluation of regimen; LOS and SSI incidence. | Adherence to international guidelines before and after intervention. | Regimen: 37.6 *vs* 91%  SSI: 32.7 *vs* 15.2%  LOS: 7.8 (5.7) *vs* 6.5 (5.6) days [mean (SD)] |
| CIED: cardiovascular implantable electronic device, LOS: length of stay, SAP: surgical antibiotic prophylaxis, SSI: surgical site infections. | | | | | |
